# First-generation Turkish immigrants' views and preferences on cardiovascular disease prevention in primary care - a qualitative study in the Netherlands

**DOI:** 10.1016/j.jmh.2025.100367

**Published:** 2025-09-26

**Authors:** Joshua A.N. van Apeldoorn, Julie S. Jansen, Eva L. Liefhebber, Özgül Uysal-Bozkir, Edanur Sert, Ralf E. Harskamp, Charles Agyemang, Edo Richard, Eric P. Moll van Charante

**Affiliations:** a Department of Public and Occupational Health, Amsterdam UMC, University of Amsterdam, Amsterdam Public Health Research Institute, Amsterdam, The Netherlands; b Department of General Practice, Amsterdam Public Health Research Institute, Amsterdam UMC, University of Amsterdam, Amsterdam, The Netherlands; c Department of Psychology, Education and Child Studies, Erasmus School of Social and Behavioural Sciences, Erasmus University Rotterdam, Rotterdam, Netherlands; d Department of Medicine, Division of Endocrinology, Diabetes and Metabolism, The Johns Hopkins University School of Medicine, Baltimore, MD, USA; e Department of Neurology, Donders Institute for Brain, Cognition and Behaviour, Radboud University Medical Centre, Nijmegen, Netherlands

**Keywords:** Cardiovascular prevention, Migrants, Turkish, Primary care, Qualitative study

## Abstract

**Objectives:**

First-generation Turkish migrants in the Netherlands face higher cardiovascular risk and are disproportionately affected by cardiovascular disease (CVD) compared to the Dutch host population. To improve prevention in primary care, we explored their views and preferences on cardiovascular prevention.

**Design:**

We conducted a qualitative study by interviewing first-generation Turkish migrants in The Netherlands. Semi-structured interviews and focus groups, conducted in Dutch or Turkish, were analyzed using thematic analysis.

**Results:**

We conducted 26 individual interviews and two sex-stratified focus group sessions. Participants were aware of CVD risk factors and related health hazards but struggled to adopt a healthy lifestyle, as family obligations, household responsibilities, and work often took precedence over personal health. All participants identified language barriers as a significant challenge, but opinions varied on whether it was necessary for GPs to understand Turkish culture. Some felt this was unnecessary, viewing GPs primarily as medical decision-makers or intermediaries for referrals to other (para)medics, with lifestyle advice outside their professional scope. They emphasized that GPs should ask openly about lifestyle rather than assuming that behaviours associated with a Turkish cultural background play a role.

**Conclusions:**

First-generation Turkish migrants in the Netherlands were aware of CVD risk, but personal responsibilities posed challenges in adopting a healthy lifestyle. Although views on the importance of GPs understanding Turkish culture varied, participants agreed that GPs should ask openly about lifestyle rather than assuming cultural relevance in cardiovascular prevention.

## 1 Introduction

Cardiovascular disease (CVD) remains a leading cause of mortality and morbidity in Europe, despite advances in preventive and therapeutic interventions (Roth et al., 2020). In Europe, most ethnic minority groups face higher rates of CVD and its risk factors, often starting at a younger age than the host populations (Modesti et al., 2016; Perini et al., 2018; Kist et al., 2021). In the Netherlands, Dutch people with a Turkish background constitute the largest migrant group. In the 1960s and 1970s, Turkish men primarily migrated to the Netherlands for work, while women mainly arrived for family reunification, with most originating from rural areas (Schellingerhout, 2004). Compared to the Dutch majority, they tend to have lower education levels and, despite their long residence in the Netherlands, experience a relatively large distance from Dutch society (Uitewaal et al., 2004). Compared to the Dutch host population, Turkish migrants have higher rates of CVD (van Oeffelen et al., 2013; Van Oeffelen et al., 2014), and its risk factors (Uitewaal et al., 2004). Following CVD and hospitalisation, they also experience higher mortality rates (Agyemang et al., 2009), and benefit less from cardiac rehabilitation (Sloots et al., 2012). Possible explanations for these disparities include: relatively high rates of low socio-economic status, higher smoking rates among Turkish men, linguistic barriers, lower access to healthcare, and cultural habits influencing diet and physical activity (Edelman et al., 2009; Jansen et al., 2011; Nicolaou et al., 2009; Jager et al., 2019). As this first-generation of migrants ages, a growing number is expected to face chronic diseases and increased healthcare needs (Centraal Bureau voor Statistiek 2020).

With the current knowledge of these health disparities, a different approach to cardiovascular prevention may benefit Turkish migrants in the Netherlands. For example, the central role of food in their culture can lead to overeating, contributing to obesity (Nicolaou et al., 2009). Turkish migrants with diabetes report that cultural factors hinder integrating lifestyle advice into daily life (Jansen et al., 2011), and that they prefer advice from professionals familiar with their eating habits (Bukman et al., 2016). In line with this, tailored lifestyle interventions have shown success in reducing waist circumference reduction and in attaining a healthy lifestyle through group meetings (Bukman et al., 2017; Teuscher et al., 2015). However, Dutch GPs have varying opinions on the necessity of culturally tailored care, with many favouring an individual approach to avoid generalization or stigmatization based on ethnic background (van Apeldoorn et al., 2023). It remains unclear how much cultural knowledge GPs need, according to their Turkish patients. Therefore, we explored first-generation Turkish migrants’ perspectives on CVD prevention in primary care, focusing on their views on risk factors, as well as barriers and facilitators to a healthy lifestyle. Additionally, we examined how they perceive the GP’s role in supporting lifestyle changes, with specific attention to the importance of cultural sensitivity and preventive guidance.

## 2 Methods

### 2.1 Participants

For this study, we included only first-generation Turkish migrants and conducted semi-structured individual interviews and two focus group sessions between 2019 and 2024. Initially, participants were recruited through GPs in large cities across the Netherlands with a significant population of Dutch citizens of Turkish origin, but this yielded a limited response. Subsequently, participants were approached through community centers (e.g. mosques, neighbourhood centres, Turkish restaurants), key figures in the Turkish community, and the authors' professional networks. Interested individuals received brief information about the study by telephone, followed by written materials in Turkish or Dutch, depending on their preference. For those with limited reading proficiency, an oral explanation of the study was provided. To achieve a diverse sample, participants were purposively sampled on 1) gender, 2) number of CVD risk factors (0–2 or ≥3) or history of CVD, and 3) level of proficiency in the Dutch language. Data were collected at home, in community centers, or, in some cases (n = 3), via telephone or video calling for participants’ convenience. During the interviews and focus groups, only the interviewees and the interviewers were present. The sessions were held at community centers with existing groups (Green and Thorogood, 2018) that meet weekly and engage in activities together. Focus groups were organised because, during data collection, we noticed that several participants stated that Dutch GPs did not need knowledge of Turkish culture. This was an unexpected finding, as cultural knowledge was initially considered a potentially important factor in improving lifestyle guidance. We hypothesised that this response might have been influenced by social desirability bias when participants interacted with ethnically Dutch interviewers who were also doctors. To explore this topic further, and with the expectation that potential bias would be reduced in a peer setting, we organised two additional sex-stratified focus groups.

### 2.2 Data collection

The individual interviews were conducted by JvA, JJ, and EL. The first focus group was conducted by ES and ÖU, both researchers with a Turkish background, while the second was led by JvA and JJ, with support from a community center group facilitator who assisted with translation and moderating the discussion. All interviewers were experienced in qualitative research, and except for JvA, female. We employed Turkish facilitators in the focus groups to minimize socially desirable responses, suspecting that awareness of the interviewers being MDs might influence participants’ responses on whether GPs need specific knowledge of Turkish culture. The focus groups were sex-separated, as a mixed-sex group could inhibit participants’ willingness to speak openly due to cultural or religious factors (Nicolaou et al., 2009). Prior to the focus groups, JJ and JvA visited the groups to build trust and explain the research purpose. The topic list (Supplementary file 1) was developed by JvA, JJ, EL, and EM, with the input from other researchers experienced in qualitative research with minority groups, as well as (para)medical colleagues of Turkish background (ÖU, ES) for accurate translation and clarification of concepts. Using open-ended questions, the interview focused on three main topics: 1) CVD and its risk factors, 2) adopting a healthy lifestyle, and 3) experiences with and expectations of Dutch GPs in providing lifestyle behaviour guidance. We iteratively adapted the interview guide during the data collection phase, based on new insights obtained during the interviews. No repeat interviews were deemed necessary. Interviews lasted between 35 and 70 min, while focus groups lasted 2 h with a 15-minute break. We determined our sample size by reaching data saturation, defined as the point when we gathered enough data to understand participants’ experiences and views, and no new themes emerged (Hennink et al., 2017). During the interviews and focus groups, we sought feedback to ensure we accurately understood participants’ views. After the focus groups, we requested feedback from participants at the community centers regarding our findings.

### 2.3 Coding and analysis

Interviews were audiotaped, transcribed verbatim and thematically analysed using the six-phase method described by Braun and Clark, with the assistance of the qualitative data analysis software MAXQDA (Braun and Clarke, 2006; Software V 2019). First, for interviews conducted in Turkish, bilingual medical students translated the transcripts into Dutch, while those conducted in Dutch were transcribed verbatim by JvA, JJ, EL or ES. By transcribing and reading transcripts the authors familiarised themselves with the collected data. Second, the initial transcripts were coded, and themes were derived from the data by JvA, JJ, and EL, who systematically reviewed the materials. All transcripts were double coded by at least two of the three researchers (JJ, JvA, and EL). Codes were discussed with EM to explore the findings and build a code tree, with discussions continuing until consensus was reached. Third, the codes were organised into potential themes, until agreement on themes was reached (JvA, JJ, EM). Fourth, thematic maps were created to represent the data after reviewing the collected material. Fifth, the final themes were repolished and structured into a narrative format. Sixth, final analysis was conducted to answer the research questions, including the selection and translation of the best descriptive quotes into English. This study was reported according to the Consolidated Criteria for Reporting Qualitative studies (COREQ) in Supplemental Table 1 (Tong et al., 2007).

### 2.4 Ethical considerations

The Medical Ethics Committee at the Amsterdam UMC (location AMC) reviewed the protocol. It stated that the Medical Research Involving Human Subjects Act (WMO) does not apply to this study (reference W19_409#19.475). Informed consent, available in Turkish and Dutch, was obtained from all participants before the start of each interview and focus group.

## 3 Results

We conducted 26 individual in-depth interviews and 2 focus groups sessions (FGS) with 10 women and 9 men, respectively, all of whom were different from the participants in the interviews. Participant characteristics are described in Table 1. The mean age of participants was 60.6 years for the interviews (range: 46–80), 55.3 years for the women's FGS (range: 49–62), and 76 years for the men's FGS (range: 64–89).

**Thematic analysis**
  We identified three key themes: perceptions of cardiovascular health, experiences of working on a healthy lifestyle, and expectations of lifestyle behaviour guidance by the GP

**Table 1:** Demographic characteristics of participants in-depth interviews (IDI) and focus group sessions (FGS).

Characteristics	IDI (*n* = 26)	FGS women	FGS men
Sex			
Female Male	10 16	10 -	- 9
Age (years)			
40–49y 50–59y 60–69y ≥70y Unknown	2 (8 %) 11 (42 %) 8 (31 %) 5 (19 %) -	1 (10 %) 8 (80 %) 1 (10 %) - -	- - 2 (22 %) 5 (56 %) 2 (22 %)
Mean age (years)	60,6 (46–80)	55,3 (49–62)	76 (64–89)
Mean duration of stay (years)	35,0	34,4	52,3
Language proficiency^b^			
Low Intermediate High Unknown	9 (35 %) 5 (19 %) 12 (46 %) -	1 (10 %) 4 (40 %) 5 (50 %) -	3 (33 %) 5 (55 %) 1 (11 %) -
History of CVD^c^	9 (35 %)	0 (0 %)	1 (11 %)
Number of CVD risk factors			
0–1 ≥ 2 Unknown	7 (25 %) 19 (70 %) -	4 (40 %) 5 (50 %) 1 (10 %)	- - 9 (100 %)*

CVD: Cardiovascular disease.

a Basic: no, lower primary or lower secondary education.

Intermediate: upper secondary or post-secondary, non-tertiary education.

High: first- or second-stage tertiary education.

b Defined as the ability to conduct the interview in Dutch.

Low: unable to understand and/or answer basic questions in Dutch.

Intermediate: basic questions could be answered in Dutch, but Turkish language was needed to clarify. High: the entire interview could be conducted in Dutch without an interpreter.

c History of CVD and risk factors were self-reported.

* Data were not gathered at the end of the FGS as participants had other appointments and left. This data could not be collected afterwards as many had already left for a long vacation in Turkey.

### 3.1 Key theme 1: perceptions of cardiovascular health

Overall, participants were well aware of CVD risk factors and used descriptions consistent with widely accepted health concepts. Risk factors, such as obesity, hypertension, diabetes, and high cholesterol were attributed to an unhealthy lifestyle, particularly poor eating habits. Specifically, the use of “the three whites” - salt, sugar, and flour - was considered unhealthy.

*“We should distance ourselves from the three whites: salt, sugar, and flour. But we don't do that.” (P15, male)*

Stress was commonly identified as a barrier to self-care and a contributor to cardiovascular risk factors, particularly hypertension. Participants with personal or family experience of CVD expressed heightened concern, fear of future events, and awareness of the biological mechanisms and lifestyle impacts on CVD. While most participants believed they had some control over their cardiovascular health, others felt limited in their influence, attributing their risk to family history or viewing it as predetermined by a higher power, making it something they had to accept.

*'It is something that can happen and then I accept it. It is something from God, so I just have to live with it.' (P7, female)*

### 3.2 Key theme 2: experiences of working on a healthy lifestyle

Overall interviewees felt that many cardiovascular risk factors were amenable to change and mentioned several facilitators and barriers in pursuing a healthy lifestyle.

#### 3.2.1 Facilitators

Many participants emphasised that the motivation to change lifestyle behaviours must come from within. This sense of personal responsibility could drive individuals to actively adopt healthier practices. In their view, religion -primarily Islam-encourage taking responsibility for their health and self-care.

*'Health and Islam are positively intertwined. […] Not eating too much, more exercise, working more often. This is what the Islam teaches us, what our Prophet tells us. People who do that, stay healthy.' (P12, female)*

Noticing the positive effects of lifestyle changes motivated participants to maintain these habits, adhere to medication, and quit smoking. Having witnessed a close relative suffer the consequences of an unhealthy lifestyle greatly motivated some participants to adopt a healthier lifestyle.

*'The dialysis of my husband was a terrible process. I don't want to end up like that; that is a good motivator for me.’ (P14, female)*

#### 3.2.2 Barriers

Family obligations, household responsibilities or work often hindered participants’ internal motivation to adopt a healthy lifestyle. Female participants mentioned that duties like cooking, cleaning, and maintaining their social circle often took precedence over their own health. One female participant gave an example why maintaining a healthy lifestyle can be especially challenging for Turkish women:

*‘The reason she cannot exercise and is gaining weight is because of all the responsibilities she takes on. It’s because she puts herself second. She has to cook, prepare breakfast, the children need to go to school, the laundry has to be done, and when is she supposed to find time for herself?’ (female FGS)*

Regarding physical activity, participants mentioned that they did not grow up with the habit of engaging in sports. As a result, taking up sports later in life felt like a significant challenge, as their established way of life made it difficult to incorporate new routines. Cultural habits regarding meals were frequently mentioned as barriers to adopting a healthy lifestyle, particularly within Turkish food culture. Sharing meals and hosting guests are highly valued, fostering connection and hospitality. These gatherings often feature abundant, high-calorie foods like baklava. Declining food is seen as disrespectful, so participants often relaxed dietary restrictions when eating with friends or family.

*'Within our culture, you always have a lot of guests. You come to my place, I come to yours. If you are not eating, that is considered impolite.' (P1, male)*

Some participants mentioned that Turkish migrants in the Netherlands come from different regions of Turkey, each with distinct dietary habits.

*‘For instance, Konya or Ankara. Over there, there is a lot of agriculture, sheep and meat. […] When I don't eat meat, I keep feeling hungry. This is the way I was raised.’ (P23, male)*

### 3.3 Key theme 3: expectations of lifestyle behaviour guidance by the GP

#### 3.3.1 GP’s role and shared decision making

Most participants viewed the GP’s role as that of a medical decision maker and an intermediary for referrals to other (para)medics, placing detailed lifestyle advice outside the GP’s professional scope and instead within the roles of physiotherapists, dieticians, or practice nurses. Participants noted that in Turkish culture, doctors are seen as authorities responsible for making decisions. When doctors ask patients which treatment option they prefer, it can make patients feel as though they are not being taken seriously or that their doctor is not sufficiently qualified.

*‘A lot of people have great respect for the expert, and they want you to take the lead. They don't want the initiative to be put back to them.' (P20, male)*

However, some participants nuanced this view, seeing shared decision-making about lifestyle behaviour as an opportunity to express their thoughts and beliefs, rather than simply allowing the GP decide for them. They often felt that they knew themselves best and should have a say in the decision-making process, which would increase their sense of autonomy.

*‘Most of the time I don't know what is in the medication, which antihypertensive has which effect. (Through discussing options) at least you're more aware. More aware and more involved.' (P21, male)*

When asked for suggestions to improve primary care, some participants mentioned that their GP should take the time to conduct yearly check-ups, such as measuring blood pressure or performing blood tests. In line with this, more frequent follow-ups after starting lifestyle changes, such as following a diet, could motivate patients to commit to a healthier lifestyle and enhance their sense of involvement from their doctor. However, many acknowledged that this may not be feasible due to the heavy work-load most GPs face.

#### 3.3.2 Language barriers

Language barriers were considered the most significant issue in communication with GPs. The few participants who had a Turkish-speaking GP highlighted that being able to speak in their mother tongue was a major advantage. The use of informal translators, such as family members, was often mentioned and considered particularly important for elderly individuals and those with lower levels of education. People in these groups may feel embarrassed if they do not understand questions or explanations, causing them to hesitate in raising their concerns.

*'Yes, I think it is a barrier for me to call the GP because I don't speak the language and don't understand it. If a doctor would call me with the assistance of a translator, that would help a lot, then the barrier to talk to the GP is taken away.' (P26, male)*

#### 3.3.4 Cultural sensitivity

For most participants, the influence of lifestyle factors related to Turkish culture on cardiovascular prevention—such as communal eating and specific food preparation methods— was not straightforward, as they did not perceive their lifestyle to differ significantly from the Dutch lifestyle. Participants emphasised that instead of assuming that a Turkish background necessitates tailored treatment, GPs should adopt a personalised approach and inquire whether cultural factors influence lifestyle choices to prevent overgeneralisation.

*'No, that (cultural distinction), is not necessary, the GP has nothing to do with that. The GP should just be there for people, their health and pay attention to that, doesn't matter where people come from.' (P22, male)*

Upon further explanation and examples provided during the interviews, some participants mentioned that it would be appreciated if GPs were aware of the importance of social cohesion in Turkish culture. Some participants mentioned that GP’s understanding of Turkish ethnicity could improve healthcare, i.e. knowledge of Turkish culture could enable dietary advice tailored to Turkish cuisine.

*‘I understand what the doctor tells me when he says: Eat less salt or fat. But it’s nice if the doctor knows a bit more about Turkish cuisine.’ (P2, male)*

Some women mentioned that it would be helpful for the GP to be aware of group activities in the neighbourhood with a culturally specific context, such as walking groups for women with Islamic religion. Attending group exercise classes served as a good incentive to continue exercising. Additionally, these initiatives provided social interaction and made physical activity enjoyable, offering opportunities to catch up with others.

*'It (physical activity group lessons) makes me happy! I really look forward to it, to be there with the group. […] We all became good friends, we tell each other everything.' (P8, female)*

## 4 Discussion

First-generation Turkish migrants in the Netherlands are well aware of CVD risk factors and possible health hazards relating to CVD, but struggle to adopt a healthy lifestyle as obligations towards family, household responsibilities, and work often took precedence, leaving personal health as a lower priority. Cultural factors play a role, as sharing traditional meals and accepting offered food when visiting friends or family were seen as essential for maintaining relationships. All participants mentioned that language barriers impeding communication with the GP when addressing lifestyle behaviours or preventative medication, are a major challenge to overcome. Regular check-ups were suggested to improve healthcare. Some participants prefer a directive approach by their GP to avoid having to take the initiative while others appreciate shared decision-making to increase autonomy. Participants had varying opinions on the knowledge GPs should have about the Turkish culture to facilitate appropriate lifestyle guidance. Some felt this was unnecessary, viewing GPs primarily as medical decision-makers or intermediaries for referrals to other (para)medics, with lifestyle advice outside their professional scope. They emphasized that GPs should ask openly about lifestyle rather than assuming that behaviours associated with Turkish cultural background play a role.

Consistent with findings among Turkish migrants with diabetes in Germany, participants felt that a healthy lifestyle was potentially beneficial in reducing CVD risk factors and events (Yilmaz-Aslan et al., 2014). Having experienced a CVD event themselves, or the experience of a relative who had gone through one, often explained their knowledge about symptoms and understanding of the severity of possible complications. Religion influenced participants' health behaviours in different ways, consistent with previous research (Yilmaz-Aslan et al., 2014; Peeters et al., 2015; Kampf and Göksu, 2014; Groenenberg et al., 2015). Most viewed it as a motivator, following the Quran's emphasis on health responsibility, while a few believed illness was divinely determined and therefore saw less of a role for themselves in prevention. Participants identified several barriers and enablers in achieving a healthy lifestyle. Limited proficiency in Dutch remains a significant barrier to effective communication with GPs regarding lifestyle advice (Bukman et al., 2016; Scheppers, 2006). A recent qualitative study found that Dutch GPs share this concern (van Apeldoorn et al., 2023). Practical barriers such as lack of time or motivation to do physical exercise are known to withhold CVD patients from exercising (Romeike et al., 2016; Alharbi et al., 2017). Prioritising family obligations, household responsibilities or work often hindering participants’ internal motivation to adopt a healthy lifestyle, aligns with previous research findings (Jansen et al., 2011; Yilmaz-Aslan et al., 2014; Teuscher et al., 2015). Furthermore, the moderation of the 'three whites' (e.g., salt, sugar, and flour) was frequently emphasised. This aligns with a previous report, which offered similar insights into how food hospitality is expressed and the difficulty in refusing abundant dishes to avoid displeasing the host (Nicolaou et al., 2009). Strategies to avoid overeating in such situations include trying only a few bites or politely indicating that one is already full, as has also been suggested in previous publications (Romeike et al., 2016).

Consistent with our findings, language concordance has been described as the main explanation for preferring ethnically-matched care providers (Bukman et al., 2016). Therefore, providing readily available official interpreters could be a crucial step toward improving cardiovascular prevention for ethnic minority populations in general practice. Some participants felt that a GP’s knowledge of Turkish culture could enhance tailored lifestyle guidance, but most did not believe their GP needed specific knowledge of Turkish culture. It is possible that participants felt that providing tailored lifestyle advice is beyond the GP’s scope, with GPs often seen as intermediaries who refer patients to dieticians or physiotherapists for such advice. Alternatively, it is possible that interviewees who provided this response thought it was the socially desirable answer to give to ethnically Dutch interviewers, whom they knew were doctors. To explore this further, we organised focus groups with bicultural translators. However, the finding remained consistent across both focus groups, despite age differences between men and women. At the end of the second focus group, a participant remarked: “You know, we are Dutch too.” This statement brought a sense of unease to the researchers and, more importantly, provided insight that an overemphasis on ethnic diversity may create a feeling of “otherness” (Conkova and Lindenberg, 2020; Johnson et al., 2004), while lifestyle differences between Turkish migrants and the Dutch population may be fewer than expected (Romeike et al., 2016; Teuscher et al., 2015). Consistent with Dutch GPs views, most participants emphasized that GPs should focus more on personal circumstances, such as living situations and family dynamics, rather than general Turkish culture (van Apeldoorn et al., 2023). The manifestations of this culture are highly diverse within the Turkish population, varying significantly in terms of religion, language and cuisine, as noted by the participants. Alternatively, participants may not have fully appreciated the influence of culture on lifestyle - and consequently on cardiovascular prevention - potentially perceiving it as an overemphasis on differences.

### 4.1 Strengths and limitations

A strength of our research is that, in addition to conducting individual interviews, we organised sex-stratified focus groups to achieve data saturation on topics that may have been susceptible to socially desirable responses. The moderators of the focus groups shared the same ethnic background as the participants, fostering relaxed conversations without language barriers. As a potential limitation, almost all data gathering was conducted in Amsterdam, and we cannot fully rule out the possibility that additional opinions or experiences may have been missed, potentially affecting the generalisability to other areas.

### 4.2 Implications for clinical practice and recommendations for future research

In clinical practice, it is advisable to ask openly, rather than assume that a Turkish background influences lifestyle or necessitates tailored advice. This potential need can be addressed directly by GPs or practice nurses who are confident in providing culturally tailored lifestyle interventions, or through collaboration with ethnically matched dieticians or community groups focused on healthy living. General practitioners should be aware that shared decision-making may benefit from a more directive approach in certain cultural contexts. However, given the differences in views on participation in decision-making observed in our study, we advise/recommend that GPs first inquire about patients’ preferences for shared decision-making before determining their approach. Working with interpreters may help bridge communication gaps and enhance patient engagement. When developing intervention studies for individuals with a Turkish background, it is recommended to offer culturally sensitive advice as an option rather than as the default. Further studies are needed to assess whether these expectations remain relevant for later generations and to explore the impact of interpreters or ethnically matched healthcare professionals on improving care and reducing health disparities.

## 5 Conclusion

First-generation Turkish migrants in the Netherlands are well aware of CVD risks but struggle to maintain a healthy lifestyle, often prioritizing family and work over personal health. The Dutch GP’s focus on shared decision-making and self-management sometimes clashes with preferences for a more directive approach and beliefs that CVD is inevitable. Participants highlighted language barriers as a major obstacle in communication, suggesting that access to translators could enhance prevention efforts. While opinions on the need for GPs to understand Turkish culture vary, it is important for GPs to ask about cultural relevance rather than assume it in cardiovascular care.

## Funding

Funding from this study came from the Netherlands Organisation for Health Research and Development (ZonMw) grant numbers: 1051003210004 and 10060011910004.

*Data availability:* The data underlying this article cannot be shared publicly due to the privacy of individuals participating in the study.

## CRediT authorship contribution statement

**Joshua A.N. van Apeldoorn:** Writing – original draft, Investigation, Conceptualization, Methodology, Formal analysis. **Julie S. Jansen:** Formal analysis, Writing – original draft, Conceptualization. **Eva L. Liefhebber:** Writing – review & editing, Conceptualization, Formal analysis. **Özgül Uysal-Bozkir:** Writing – review & editing. **Edanur Sert:** Writing – review & editing. **Ralf E. Harskamp:** Writing – review & editing. **Charles Agyemang:** Writing – review & editing. **Edo Richard:** Writing – review & editing. **Eric P. Moll van Charante:** Supervision, Writing – review & editing, Conceptualization.

## Declaration of competing interest

The authors declare that they have no known competing financial interests or personal relationships that could have appeared to influence the work reported in this paper.

## Appendix Supplementary materials

Image, application 1

Image, application 2

## References

[bib0001] Agyemang C., Vaartjes I., Bots M.L., van Valkengoed I.G., de Munter J.S., de Bruin A. (2009). Risk of death after first admission for cardiovascular diseases by country of birth in The Netherlands: a nationwide record-linked retrospective cohort study. Heart..

[bib0002] Alharbi M., Gallagher R., Neubeck L., Bauman A., Prebill G., Kirkness A., Randall S. (2017). Exercise barriers and the relationship to self-efficacy for exercise over 12 months of a lifestyle-change program for people with heart disease and/or diabetes. Eur. J. Cardiovasc. Nurs..

[bib0003] Braun V., Clarke V. (2006). Using thematic analysis in psychology. Qual. Res. Psychol..

[bib0004] Bukman A.J., Teuscher D., Ben Meftah J., Groenenberg I., Crone M.R., van Dijk S. (2016). Exploring strategies to reach individuals of Turkish and Moroccan origin for health checks and lifestyle advice: a mixed-methods study. BMC. Fam. Pract..

[bib0005] Bukman A.J., Teuscher D., Meershoek A., Renes R.J., van Baak M.A., Feskens E.J. (2017). Effectiveness of the MetSLIM lifestyle intervention targeting individuals of low socio-economic status and different ethnic origins with elevated waist-to-height ratio. Public Health Nutr..

[bib0006] Centraal Bureau voor Statistiek (2020).

[bib0007] Conkova N., Lindenberg J. (2020). The experience of aging and perceptions of "aging well" among older migrants in the Netherlands. Gerontologist.

[bib0008] Edelman D., Christian A., Mosca L. (2009). Association of acculturation status with beliefs, barriers, and perceptions related to cardiovascular disease prevention among racial and ethnic minorities. J. Transcult. Nurs..

[bib0009] Green J., Thorogood N. (2018).

[bib0010] Groenenberg I., Crone M.R., van Dijk S., Gebhardt W.A., Ben Meftah J., Middelkoop B.J. (2015). Check it out!' Decision-making of vulnerable groups about participation in a two-stage cardiometabolic health check: a qualitative study. Patient. Educ. Couns..

[bib0011] Hennink M.M., Kaiser B.N., Marconi V.C. (2017). Code saturation versus meaning saturation: how many interviews are enough?. Qual. Health Res..

[bib0012] Jager M.J., van der Sande R., Essink-Bot M.-L., van den Muijsenbergh M.E.T.C (2019). Views and experiences of ethnic minority diabetes patients on dietetic care in the Netherlands – a qualitative study. Eur. J. Public Health.

[bib0013] Jansen Y., Uitewaal P.J.M., Wijsman-Grootendorst A., Geelhoed-Duijvestijn P. (2011). Sociale en culturele problemen bij het opvolgen van leefstijladviezen door allochtone diabetici [Social and cultural problems in the compliance with lifestyle advice by immigrant diabetics]. Ned. Tijdschr Geneesknd.

[bib0014] Johnson J.L., Bottorff J.L., Browne A.J., Grewal S., Hilton B.A., Clarke H. (2004). Othering and being othered in the context of health care services. Health Commun..

[bib0015] Kampf A., Göksu A. (2014). Perceptions of risk and preventative strategies with respect to cardiovascular diseases in people of Turkish migrant background in Germany: findings of a pilot study. Crit. Public Health.

[bib0016] Kist J.M., Smit G.W.G., Mairuhu A.T.A., Struijs J.N., Vos R.C., van Peet P.G. (2021). Large health disparities in cardiovascular death in men and women, by ethnicity and socioeconomic status in an urban based population cohort. EClinicalMedicine.

[bib0017] Modesti P.A., Reboldi G., Cappuccio F.P., Agyemang C., Remuzzi G., Rapi S. (2016). Panethnic differences in blood pressure in Europe: a systematic review and meta-analysis. PLoS. One.

[bib0018] Nicolaou M., Doak C.M., van Dam R.M., Brug J., Stronks K., Seidell J.C. (2009). Cultural and social influences on food consumption in Dutch residents of Turkish and Moroccan origin: a qualitative study. J. Nutr. Educ. Behav..

[bib0019] Peeters B., Van Tongelen I., Duran Z., Yuksel G., Mehuys E., Willems S. (2015). Understanding medication adherence among patients of Turkish descent with type 2 diabetes: a qualitative study. Ethn. Health.

[bib0020] Perini W., Snijder M.B., Peters R.J.G., Kunst A.E. (2018). Ethnic disparities in estimated cardiovascular disease risk in Amsterdam, the Netherlands: the HELIUS study. Neth. Heart. J..

[bib0021] Romeike K., Abidi L., Lechner L., de Vries H., Oenema A. (2016). Similarities and differences in underlying beliefs of socio-cognitive factors related to diet and physical activity in lower-educated Dutch, Turkish, and Moroccan adults in the Netherlands: a focus group study. BMC. Public Health.

[bib0022] Roth G.A., Mensah G.A., CO Johnson, Addolorato G., Ammirati E., Baddour L.M. (2020). Global burden of cardiovascular diseases and Risk factors, 1990-2019: update from the GBD 2019 study. J. Am. Coll. Cardiol..

[bib0023] Schellingerhout R. Gezondheid en welzijn van allochtone ouderen. 2004.

[bib0024] Scheppers E. (2006). Potential barriers to the use of health services among ethnic minorities: a review. Fam. Pract..

[bib0025] Sloots M., Bartels E.A., Angenot E.L., Geertzen J.H., Dekker J. (2012). Adapted cardiac rehabilitation programme to improve uptake in patients of Moroccan and Turkish origin in The Netherlands: a qualitative study. J. Clin. Nurs..

[bib0026] Software V (2019).

[bib0027] Teuscher D., Bukman A.J., Meershoek A., Renes R.J., Feskens E.J.M., van Baak M.A. (2015). Adapting an effective lifestyle intervention towards individuals with low socioeconomic status of different ethnic origins: the design of the MetSLIM study. BMC. Public Health.

[bib0028] Teuscher D., Bukman A.J., van Baak M.A., Feskens E.J.M., Renes R.J., Meershoek A. (2015). Challenges of a healthy lifestyle for socially disadvantaged people of Dutch, Moroccan and Turkish origin in the Netherlands: a focus group study. Crit. Public Health.

[bib0029] Tong A., Sainsbury P., Craig J. (2007). Consolidated criteria for reporting qualitative research (COREQ): a 32-item checklist for interviews and focus groups. Int. J. Quality in Health Care.

[bib0030] Uitewaal P., Bruijnzeels M., De Hoop T., Hoes A., Thomas S. (2004). Feasibility of diabetes peer education for Turkish type 2 diabetes patients in Dutch general practice. Patient. Educ. Couns..

[bib0031] Uitewaal P.J., Goudswaard A.N., Ubink-Veltmaat L.J., Bruijnzeels M.A., Hoes A.W., Thomas S. (2004). Cardiovascular risk factors in Turkish immigrants with type 2 diabetes mellitus: comparison with Dutch patients. Eur. J. Epidemiol..

[bib0032] van Apeldoorn J.A.N., Roozekrans A.K., Harskamp R.E., Richard E., Agyemang C., Moll van Charante E.P (2023). General practitioners' views on cardiovascular prevention for ethnic minorities-a qualitative study in the Netherlands. Fam. Pract..

[bib0033] Van Oeffelen A.A., Agyemang C., Stronks K., Bots M.L., Vaartjes I. (2014). Incidence of first acute myocardial infarction over time specific for age, sex, and country of birth. Neth. J. Med..

[bib0034] van Oeffelen A.A.M., Vaartjes I., Stronks K., Bots M.L., Agyemang C. (2013). Incidence of acute myocardial infarction in first and second generation minority groups: does the second generation converge towards the majority population?. Int. J. Cardiol..

[bib0035] Yilmaz-Aslan Y., Brzoska P., Bluhm M., Aslan A., Razum O. (2014). Illness perceptions in Turkish migrants with diabetes: a qualitative study. Chronic. Illn..

