# Supplementary material for: First-generation Turkish immigrants' views and preferences on cardiovascular disease prevention in primary care - a qualitative study in the Netherlands
Source: J Migr Health. 2025 Sep 26;12:100367. doi: 10.1016/j.jmh.2025.100367 (PMC12517069; doi:10.1016/j.jmh.2025.100367)
Supplement: Supplementary file 1 [file mmc1.docx]

**Interview guide - First-generation Turkish Immigrants' Views and Preferences on Cardiovascular Disease Prevention in Primary Care - a Qualitative Study in the Netherlands**

**Consent to participation and recording**

**Introduction participant:**
Age, sex, country of birth, years in the Netherlands, occupation, educational attainment, living situation, married, children, religion.

**General**

- What does being "healthy" mean to you?

**Risk Factors**

- Have you ever had a heart attack or stroke, or do you have vascular problems, for example in the legs, heart, or brain?
- Are you aware that high blood pressure is dangerous? What could it lead to?
- Ask similar questions about other risk factors:
  - What is the danger of being overweight? What are the risks of not exercising enough?
  - What does sufficient exercise mean? How much exercise is healthy? Do you have enough time to exercise adequately? How much time would that take?
  - What is the danger of diabetes? How can it be treated?
  - Do you have risk factors (as mentioned above)? What do you think about this?
  - Do you believe you can influence this?
  - *If predestination is mentioned ("it's in the genes," "it's determined by Allah") → follow up: Can you influence this? How? How can the general practitioner (GP) provide the best advice in this regard?*
- Stress**:** Do you think stress plays a role in your health? Is it important to reduce it? Do you think the GP could support you in this? What advice could they give?
- Smoking**:** Do you think smoking affects your health?
- In case of smoking: have you ever been advised to quit (by a GP/practice nurse/specialist)? What was good about that advice? Was it sufficient for you?

**Healthy Lifestyle**

- Have you ever received advice to "live healthier"? What exactly did they tell you? (more exercise or healthier eating?) What did you think about it?
  - If you have never received this advice → did people you know receive advice?
  - Specify whether it concerns food/exercise → follow up on each piece of advice.
- Who gave you this advice? The general practitioner → delve deeper into GP’s advice
- Media/newspaper → where from? Dutch media, Turkish media? What's the difference?
- Regarding advice: Were you able to follow it? Why did you decide to follow it? What motivated you? What helped you stick to it? Did you do this alone or together?
- What made it difficult to follow such advice?
- Is it harder to maintain a healthy lifestyle (diet/exercise advice) when you are with family or friends? What role do they play? (Is it, for example, impolite to refuse food?) Do you think your faith or Turkish background plays a role in following a diet? If yes, in what way?
- Do you feel that lifestyle advice does not fit well with your Turkish background? What is the reason for this?

**Advice from GP/ practice nurse**

- For what complaints do you visit your GP? Would you go to the GP for lifestyle advice (quitting smoking/exercising more/eating healthier)? If you think this isn't possible → how can the GP increase awareness among patients of this possibility?
- If you have ever received advice (more exercise, healthier eating?) from the GP or practice supporter: what did you think of that advice? Did it help you? Why or why not? How could this advice be improved? Can you give an example?
- Can a GP or their staff give you good advice on lifestyle? Even if they have no knowlegde about your Turkish background?
- Should the GP (and/or practice supporter) know more about Turkish cultural lifestyle to provide good lifestyle advice? Would it be better if the GP referred you to someone with a Turkish background for this advice? If yes; what is the difference?
- Do you think advice is easier to follow if you carry it out with a group of people from the same background? For example, walking together or cooking together?

**Shared Decision-Making** **GP**

- Do you visit your GP often? Do you trust your GP?
- Does your GP always understand you well or sometimes not? What causes this?
- Do you feel that the GP is aware of your cultural background? Do you think it’s important for the GP to know this? What should the GP know about your Turkish culture to provide you with good care?
- Do you ever visit another GP/doctor in Turkey? For what problems do you consult a doctor in Turkey? What is different?
- Do you trust a Turkish doctor more than a Dutch doctor?
- What does a “good” doctor mean to you?
- How could a GP improve the care for you?

**Shared Decision-Making**

Outline the situation: The patient and doctor discuss treatment options, pros and cons, and decide together which option to pursue. This includes discussions about additional research, which treatment options (medication A or B, no medication?).

**Example Situation 1:** (only if above explanation is insufficient) Mr. A, 56 years old, visits the practice supporter of the GP for a check-up regarding high blood pressure and the risk of cardiovascular diseases. His blood pressure is slightly high, with a systolic reading of 160 (normal is 120). The high blood pressure could be a reason to start medication. Mr. A could also try to improve his lifestyle to lower his blood pressure. The practice supporter discusses which medications he might take, how often he should take them, and what the side effects might be. Mr. A and the practice nurse also discuss lifestyle changes (healthier eating, more exercise). After discussing it at home and reading the information, Mr. A decides he first wants to try lifestyle changes; coincidentally, his wife just started a new diet, and the neighbor has joined a walking group. Moreover, if his blood pressure is still high after the follow-up, he can always start medication then.

- When the GP gives you advice or prescribes medication, do you feel like you come to this advice together, or more like the GP decides for you? What do you prefer, to decide together or to have the GP decide?
- Have you ever had a similar situation with your GP? What did you think of it?
- What do you think when a GP asks you what you want?
- Do you think this improves care or not? Do you think your Turkish background and experience with Turkish doctors, where everything goes very quickly, influences this?
- Lastly: If there’s one thing your GP could improve in the care for Turkish-Dutch people of your generation, what would it be?

**Closing** Thank you. Feedback? (Were the topics covered too long/too short?)
